# Experimental modelling of failure risks using wipe dispenser systems and ready-to-use disinfecting wipes and their consequences

**DOI:** 10.3205/dgkh000571

**Published:** 2025-07-14

**Authors:** Wiebke Lüdtke, Paula Zwicker, Jürgen Gebel, Martin Exner, Axel Kramer

**Affiliations:** 1 Institute of Hygiene and Environmental Medicine, University Medicine Greifswald, Greifswald, Germany; 2 Institute for Hygiene and Public Health, University Clinics Bonn, Bonn, Germany; 3 VAH – Association for Applied Hygiene c/o Institute for Hygiene and Public Health, Bonn, Germany; 4 University Bonn, Bonn, Germany

**Keywords:** wipe dispenser systems, ready-to-use disinfectant wipes, disinfection methods, flow pack, stand-up bag, wipe saturation, failure risks

## Abstract

**Introduction:**

A blinded survey in 81 dental practices, 84 medical practices, and 35 hospitals revealed that for conducting disinfecting surface cleaning and surface disinfection, instead of reusable clothes moistened on-site with disinfectant solution, either wipe dispenser systems for self-preparation or ready-to-use (RTU) wipes are being used. Therefore, the aim of this study was to examine, i.e., the impact of incorrect loading of the wipe roll with disinfectant solution (DS) when using wipe dispenser systems, and the consistency of the DS delivery amount when using RTU wipes.

**Method:**

In two different wipe dispenser systems, the saturation of the wipe roll after loading with DS was visually inspected and photographically documented by adding 0.1% fluorescein sodium to the disinfectant solution. The coverage of the wipes used on a melamine resin surface (75x133 cm) was visually checked after a defined wiping mode following analogous staining.

For two RTU products, a flow pack and a stand-up bag pwith the opening at the top, the saturation of the wipes and the delivery amount of the DS during use were gravimetrically determined.

**Results:**

In the wipe dispenser system with alcohol-based DS, the amount of disinfectant solution released decreased when the solution was loaded horizontally or vertically, instead of circularly as recommended by the manufacturer. After circular loading with the manufacturer-recommended wetting time of 30 minutes, the wipe rolls were evenly saturated, and the delivery amount onto the surface during wiping disinfection was sufficiently constant. In the wipe dispenser system with an oxygen-releasing DS, after horizontal instead of circular loading the residual volume in the dispenser after removal of the last cloth was 320 ml instead 350 ml. The delivery amount onto the surface during wiping disinfection was therefore also lower (4.2+0.574 g instead of 5.0+0.606 g, p<0.0001).

For the flow pack, uniform saturation was achieved when the package was stored upside down with the sealed opening facing downward the night before the first use. In the vertical pack, the delivery amount of the first wipe was significantly lower than that of the subsequent wipes.

**Conclusion:**

For the tested flow pack, it should be noted in the user manual that the flow pack should be stored upside down, i.e., with the opening facing downward, for more than 12 hours before the first use, to achieve uniform wetting of all wipes.

For the stand-up bag, it is important to follow the manufacturer's instruction that the first wipe be discarded.

Since the DS delivery amount differed between the flow pack and vertical pack, it would be beneficial if, as in both cases, the manufacturer generally specified the reach for wiping disinfection for each RTU product.

## Zusammenfassung

**Einleitung:**

Eine verblindete Umfrage in 81 Zahnarztpraxen, 84 Arztpraxen und 35 Krankenhäusern ergab, dass zur Durchführung der desinfizierenden Flächenreinigung und Flächendesinfektion anstelle von Wischtüchern, die vor Ort mit Desinfektionslösung benetzt werden, zunehmend entweder Tuchspendesysteme zum Selbstansetzen oder Ready to use (RTU) Tücher verwendet werden. Daher sollten als mögliche Fehlerquellen beim Einsatz von Tuchspendesystemen die Auswirkung einer falscher Beschickung der Tuchrolle mit Desinfektionslösung (DL) und bei RTU Tüchern die Konstanz der Abgabemenge der DL während der Nutzung überprüft werden. Ergänzend sollte die Zeit bis zur Lufttrocknung der desinfizierten Fläche nach der Wischdesinfektion bestimmt werden.

**Methode:**

Bei zwei unterschiedlichen Tuchspendesystemen wurde die Durchtränkung der Tuchrolle nach Beschickung mit DL durch Zusatz von 0,1% Fluorescein-Natrium zur Desinfektionslösung visuell überprüft und fotografisch dokumentiert. Die Reichweite der auf einer Melaminharzfläche (75x133 cm) eingesetzten Wipes wurde nach festgelegtem Wischmodus nach analogem Anfärben visuell geprüft.

Bei zwei RTU Produkten, ein Flowpack und ein Standbeutel, wurden die Durchtränkung der Tücher und die Abgabemenge der DL während der Nutzung gravimetrisch bestimmt

**Ergebnisse:**

Beim Tuchspendesystem mit DL auf Alkoholbasis verringerte sich die Abgabemenge an Desinfektionslösung, wenn die Lösung statt vom Hersteller empfohlen nicht zirkulär, sondern waagerecht oder senkrecht aufgebracht wurde. Nach der vom Hersteller empfohlenen Benetzungszeit von 30 min waren die Tuchrollen gleichmäßig durchtränkt und die Abgabemenge auf die Fläche bei Wischdesinfektion war ausreichend konstant. Beim Tuchspendesystem mit DL auf Basis eines Sauerstoffabspalters betrug das Restvolumen nach Entnahme des letzten Tuchs bei horizontaler Beschickung nur 320 ml statt 350 ml nach vertikaler Beschickung. Demzufolge verrringerte sich auch die Abgabemenge bei der Wischdesinfektion (4,2+0,574 g statt 5,0+0,606 g, p<0.0001).

Beim Flowpack wurde die gleichmäßige Durchtränkung erreicht, wenn die Packung am Vorabend vor dem ersten Anbruch umgekehrt mit der verschlossenen Öffnung nach unten gelagert wurde. Beim Standbeutel war die Abgabemenge des ersten Tuchs signifikant geringer als bei den darauffolgenden Tüchern.

**Schlussfolgerung:**

Bei Tuchspendesystemen sind die Herstellerangaben zum Beschicken der Tuchrolle mit DL einzuhalten.

Beim geprüften Flowpack sollte in der Gebrauchsanweisung darauf hingewiesen werden, dass das Flowpack vor dem ersten Anbruch für mehr als 12 h umgekehrt, d.h. mit der Öffnung nach unten, gelagert werden sollte, um eine gleichmäßige Benetzung aller Tücher zu erzielen. Beim Standbeutel ist die Herstellerangabe zu beachten, dass das erste Tuch zu verwerfen ist.

Da sich die Abgabemenge der DL zwischen Flowpack und Standbeutel unterschied, wäre es vorteilhaft, wenn grundsätzlich vom Hersteller für jedes RTU Produkt die Reichweite zur Wischdesinfektion angegeben wird.

## Introduction

Disinfecting surface cleaning or surface disinfection are essential components of standard precautions [1], because environmental contamination is an independent risk factor for acquiring nosocomial pathogens [2], [3], [4], [5], [6], [7], [8], [9]. Nosocomial pathogens are primarily acquired from surfaces close to patients, especially through the hands of patients and indirectly transmitted to patients by the hands of staff [10], [11], [12], [13]. However, they can also be inhaled through turbulence from the surfaces [14]. The effectiveness of disinfecting surface cleaning, particularly of surfaces close to patients, has been demonstrated in clinically controlled studies for the prevention of healthcare-associated infections (HAI) [15], [16], [17], [18], [19], [20], [21], [22], [23]. Disinfecting surface cleaning has also proven effective in controlling nosocomial outbreaks, especially as part of intervention bundles [24], [25], [26], [27], [28], [29], [30]. For aseptic activities, surface disinfection ensures the necessary environment with reduced pathogens [31].

The traditional method of disinfecting surface cleaning or surface disinfection involving the immersion of reusable wipes in disinfectant solution has lost importance due to the labor involved in reprocessing the used wipes and the inconvenient handling, especially for small surfaces. Additionally, there is a risk of incorrect application when the cleaning tools are repeatedly dipped into the disinfectant solution. This compromises effectiveness and can lead to widespread dissemination of nosocomial pathogens.

To gain an overview of the use of wipe dispenser systems and ready-to-use (RTU) wipes, a survey was conducted in preparation for this experimental study in 200 healthcare facilities. In medical practices, spray disinfection was most used (Table 1 (Tab. 1)). It is important to note that application by spraying should only be performed in exceptional cases, such as surfaces that are otherwise inaccessible, due to the risk of inhalation and lack of cleaning effect [32]. Particularly, quaternary ammonium compounds can cause allergen- and irritant based asthma [33]. Alarmingly, the results of a prospective cohort study conducted across 14 US states found that exposure to disinfectants among female nursing staff was significantly associated with the incidence of chronic obstructive pulmonary disease, which was not influenced by smoking or asthma status [34]. The second most common systems used in medical practices were wipe dispenser systems for self-setup. In contrast, flow packs and wipes in stand-up bag or box dispensers were mainly used in hospital settings (Table 1 (Tab. 1)). No cases of immersion of reusable wipes in disinfectant solution were reported.

Since either wipe dispenser systems are used – where a wipe roll is inserted and moistened with DS – or RTU wipes are utilized, the saturation of the wipe rolls or the delivery amount of DS during the course of use of RTU products, respectively, should be gravimetrically determined.

## Method

### Wipe dispenser systems tested 

In *wipe dispenser system 1* (70 sheets per roll, sheet size 28x23 cm; manufacturer Schülke & Mayr GmbH, Norderstedt, Germany), an alcohol-based disinfectant is used (containing 25 g of ethanol (94% w/w) and 35g of propan-1-ol per 100 g). According to the manufacturer, the saturation of the wipe roll should be done by placing the roll vertically in the container and adding 1.5 liters of the disinfectant solution (DS) 30 minutes before first use. The DS should be applied slowly in a circular motion from the inside out. The shelf life is limited to 28 days. 

In *wipe dispenser system 2* (120 sheets per roll, sheet size 28x28 cm; manufacturer Antiseptika GmbH, Pulheim, Germany), the disinfectant used is an oxygen-releasing agent, potassium peroxymonosulfate sulfate (0.6 g per 100 g). The saturation process as per the manufacturer’s instructions is similar to system 1 and is limited to 14 days of use post-saturation.

As RTU products, a flow pack and a vertical pack (stand-up bag) were examined. The flow pack, based on 17.4 g of propan-2-ol and 12.6 g of ethanol (94% w/w), contains 80 sheets per pack, each sheet measuring 25x25 cm, covering approximately 1.5 m², with a shelf life of one month after opening (manufacturer Schülke & Mayr GmbH, Norderstedt, Germany). The stand-up bag, based on potassium peroxymonosulfate sulfate, contains 100 sheets per pack, each sheet measuring 30x30 cm, with a shelf life of one month after opening (manufacturer Antiseptika GmbH, Pulheim, Germany).

### Experimental investigations 

#### Preliminary experiment 

In a preliminary test conducted in a dental practice, wipe dispenser system 1 was used to determine the frequency of wipe use and when they are depleted, to establish an interval for simulating conditions in the laboratory experiments.

#### Laboratory experiments with the wipe dispenser systems 

For both systems, the saturation of the wipe rolls was compared between the manufacturer’s recommended circular application (1.5 L) with vertical and horizontal applications. After the declared saturation time (30 minutes), the saturation of the wipes was assessed based on staining, then documented photographically. The saturation was determined using Fluorescein sodium (Uranin, 98.5–100.5%, FlukaTM [35]), a yellowish dye visible to the naked eye, soluble in ethanol (up to 70g/L) and water (up to 500g/L), at a final concentration of 0.1%. The best results for assessing wetting were obtained with a 0.1% solution based on preliminary tests with 1%, 0.1%, and 0.01% solutions.

**Figure 1:**
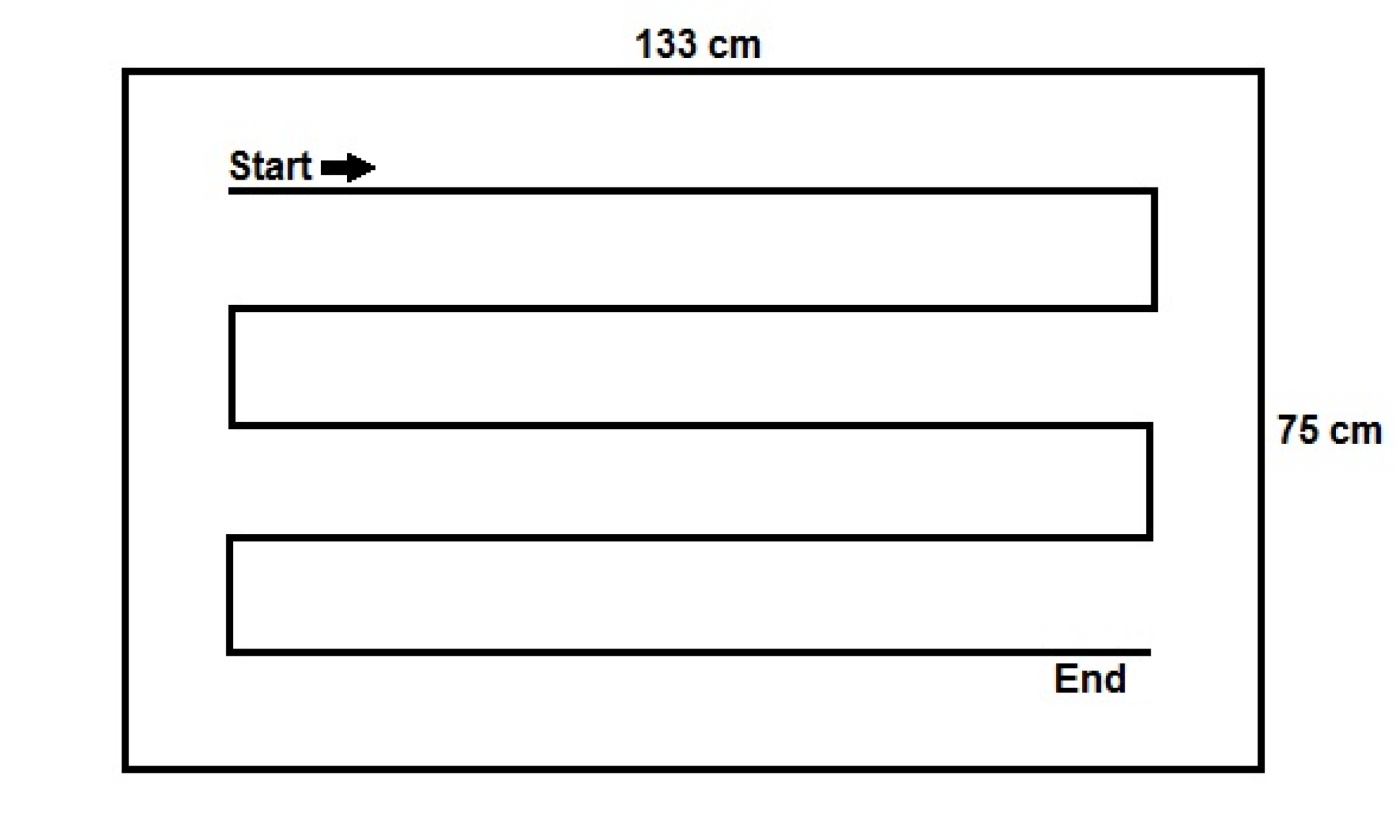
Test area and procedure of wiping disinfection process

The amount of DS released was determined by weighing after removal from the dispenser (initial weight) and after surface disinfection (final weight). After each wipe was taken, the dispenser was closed. For the wiping disinfection, the unfolded wipe was placed on a laboratory table surface covered with tape (0.9975 m²) made of melamine resin and wiped from the top left to the bottom right at a consistent speed using the flat of the hand (Figure 1 (Fig. 1)). After the last wipe was taken, the remaining liquid in the dispenser system was measured.

To determine the time until air drying on the surface, the time until visual dryness was measured for 20 wipes after the wiping process was completed (Timer ROTILABO^®^, Carl Roth GmbH+Co. KG, Karlsruhe, Germany).

#### RTU products 

The loading of the wipes with DS, their release onto the surface, and the time until air drying of the surface were determined.

The dry weight (calibrated precision scale Sartorius Basic BA 110, Sartorius AG Göttingen, Germany) was measured for 20 wipes from the wipe dispenser systems and for the RTU wipes after being stored in the air at 22°C and a relative humidity of 30±3% for 7 days.

#### Tests in a dental practice with flow packs 

All tests were conducted within the declared usage period. In trial 1, the weight of each wipe was determined after opening and consecutive removal. In trials 2–5, the flow packs were used for 6 hours each, after which 10 wipes were sequentially removed and weighed. The packs were then used for 14, 15, 20, or 23 days, and weights were measured at three time points. As the flow pack was depleted before the third measurement in trial 5, only two measurements were conducted. The flow packs were consistently stored horizontally. In trial 6, the pack was stored for 12 hours overnight with the closed opening facing downward. In trial 7, after being used for 8 hours, the pack was again stored overnight (12 hours) with the closed opening facing downward. In both cases, the wipes were sequentially removed and weighed afterwards (see Table 2 (Tab. 2)).

#### Statistical analysis 

Normal distribution was tested using the Shapiro Wilk test. Outliers were identified using the ROUT test. If the data were normally distributed, statistical significance was calculated using the t-test; if non-normally distributed, the Mann-Whitney test was used.

## Results

### Preliminary experiment 

On average, a wipe was taken every 10 minutes. Therefore, this interval was selected for the laboratory experiments with RTU wipes.

### Wipe dispenser system 1 

The dry weight per wipe was 3.3±0.09 g (n=20). 

In trial 1, after circular loading, 20 wipes were sequentially removed. The amount of DS released was 5.7±0.36 g. The amount released from the first wipe (5.5 g) did not significantly differ from the amount released from all the other wipes (Table 3 (Tab. 3)). Only wipe 19 showed a tendentially lower amount released, 4.8 g. Wipe 19 was simply “furthest from the rest”, but not an outlier. The series was normally distributed. After 264.6 s (4.4 min) ±31.85 s, the disinfected surface was visually dry. In trial 2, after circular loading, there were no significant differences in the amount released from the wipes. Wipes 10 and 11 tended to release more. In trial 3, a repeat of trial 2, the amount of DS released onto the surface was tendentially lower only for wipe 2, at 4.95 g compared to the average. The values were normally distributed. There were no outliers.

In trial 4, with vertical loading of the wipe dispenser, the release amount onto the surface for wipe 2 (3.8 g) and wipe 60 (3.9 g) was significantly lower (outliers). The distribution was non-normal. The amount of residual liquid in the wipe dispenser was 240 mL, significantly higher than the 186 mL left after circular wetting. In trial 5, with horizontal loading, the release amount from the first three wipes (3.6 g, 3.9 g, and 4.0 g), as well as from wipes 5 (4.3 g) and wipe 7 (4.5 g), was significantly lower. Throughout the series, the release amount was also three times (wipes 11 and 12 with 4.4 g, wipe 33 with 4.6 g) significantly below the average of 5.4±0.694g. The distribution was normal without outliers. The residual amount left in the wipe dispenser after all 70 wipes were used was least with circular loading (Table 3 (Tab. 3)). 

Despite some uneven wetting of wipes with horizontal and vertical loading, the average amounts of DS released during wiping did not differ from the values after circular loading (Table 3 (Tab. 3)).

### Wipe dispenser system 2 

The dry weight per wipe was 2.2±0.057 g (n=20).

In trial 1, with circular wetting, the release amount from wipe 1 (4.68 g) did not differ from the average. The distribution was normal without outliers. The drying time exceeded the measurement period of 540 seconds nine times, so no average time could be determined. The shortest time was 308 seconds. In trial 2, starting from wipe 2, the average release amount was reached (wipe 1 had 4 g) and was consistent throughout the series except for wipe 4 (2.7 g) and wipe 120 (2.7 g), both outliers. The distribution was non-normal..

In trial 3, with vertical loading, the average release amount was reached starting from wipe 1. This means all wipes released the same average amount except wipe 14 (7 g) and wipe 50 (3 g). The values were normally distributed. 

In trial 4, with horizontal wetting of the wipe roll, the average release amount was not reached until wipe 15. No outliers were identified. The values were not normally distributed.

The residual amount in the wipe dispenser was lowest at 305 mL with circular filling. With vertical filling, 320 mL were left, and with horizontal filling, 350 mL remained in the wipe dispenser (Table 4 (Tab. 4)).

### Flowpack 

The dry weight per wipe was 3.2±0.18 g.

During wipe removal at 2-minute intervals (n=20), only wipe 19 had a tendentially higher release amount (3.3 g). The values were not normally distributed but without outliers. The surface was visually dry after 101.1±24.185 seconds.

During wipe removal at 10-minute intervals, the average weight of the wipes was tendentially higher (Table 5 (Tab. 5)). The values were again not normally distributed with no outliers. In both cases, no residual liquid remained in the flow pack.

During testing in a dental practice , in trial 1, the weight increased steadily from the initial 8.59 g to 23.22 g at wipe 78 and 22.44 g at wipe 79 (wipe 80 was missing) after consecutive removal following opening (Table 2 (Tab. 2)). The values were not normally distributed and there were no outliers. After each 6-hour use , the average weight was significantly lower than when the pack was used up to 23 days. The average weights after further use for 14, 15, 20, and 23 days did not differ significantly. When the pack was stored overnight (12 hours) upside down, i.e., with the closed opening facing down, the weight at removal was not reduced (Table 2 (Tab. 2)).

#### Vertical pack (stand-up bag) 

The dry weight of the wipes was 3.6±0.11 g (n=20).

In the first trial, there were no significant deviations from the average. The distribution was normal and without outliers. Optical dryness was achieved nine times within the observation period of 9 minutes (460±79.76 seconds). Eleven times, the drying took longer than 9 minutes, so no average could be provided for the use of 20 wipes.

In the second experiment, a significantly lower amount of DS was released from the first wipe removed (1.28 g). There was a normal distribution with one outlier (wipe 1) (Table 6 (Tab. 6)).

## Discussion

### Wipe dispenser system 1 

Comparing the average values of wipe wetting from trials 1-3, it is clear that the uptake amount for the intended application was sufficiently constant after circular filling, provided that the manufacturer's prescribed wetting duration of 30 minutes was followed (Table 3 (Tab. 3)). With horizontal and vertical loading, not all wipes released the same amount, and more residual disinfectant remained after the wipe roll was used up. This confirms that it is imperative to adhere the manufacturer's prescribed circular loading.

### Wipe dispenser system 2 

While vertical loading did not have a detrimental effect, the average release amount was not reached until wipe 16 with horizontal loading, indicating that this method of loading should not be used. Tendentially, more DS was released from the wipe based on alcohol than from the one based on oxygen releasing wipes.

### Flowpack 

In the laboratory test with horizontal storage of the flow packs, no decrease in the released amount of DS was noticeable, but the values were not normally distributed. The fact that the average weigth of the wipes during removal at 10-minute intervals was tendentially higher than during sequential removal, this indicates that the saturation of the wipes occurs after a delay. This was confirmed in the practical test, where the wipes removed after each 6-hour use were significantly lighter than those used between 14 and 23 days. When the flow packs were stored overnight with the opening facing down, this was not observed. This leads to the recommendation that the flow packs should be stored upside down overnight before first use.

### Vertical pack 

In one test, the amount of DS released from Wipe 1 was significantly lower than from the following wipes. This confirms the manufacturer's recommendation that the first wipe should be discarded after opening. More DS was released from the oxygen-releasing wipe than from those based on alcohols. This suggests that it would be generally beneficial if the manufacturer specified the range of the disinfectant area for wipe disinfection.

## Conclusions

For wipe dispenser systems, the manufacturer's instructions for loading the wipe roll with DS must be followed. Otherwise, sufficient wetting is not achieved.

For the tested flow pack, the user’s manual should mention that the flow pack should be stored upside down, i.e., with the opening facing downward, for more than 12 hours before the first use, to achieve uniform wetting of all wipes.

For the vertical pack, the manufacturer's instruction that the first wipe should be discarded must be followed.

## Notes

### Competing interests

The authors declare that they have no competing interests.

### Authors’ ORCIDs

- Zwicker P: https://orcid.org/0000-0001-8891-7160
- Gebel J: https://orcid.org/0000-0001-9328-3174
- Kramer A: https://orcid.org/0000-0003-4193-2149

## Figures and Tables

**Table 1:**
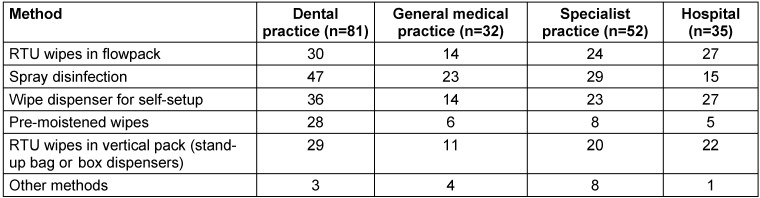
Disinfection methods used

**Table 2:**
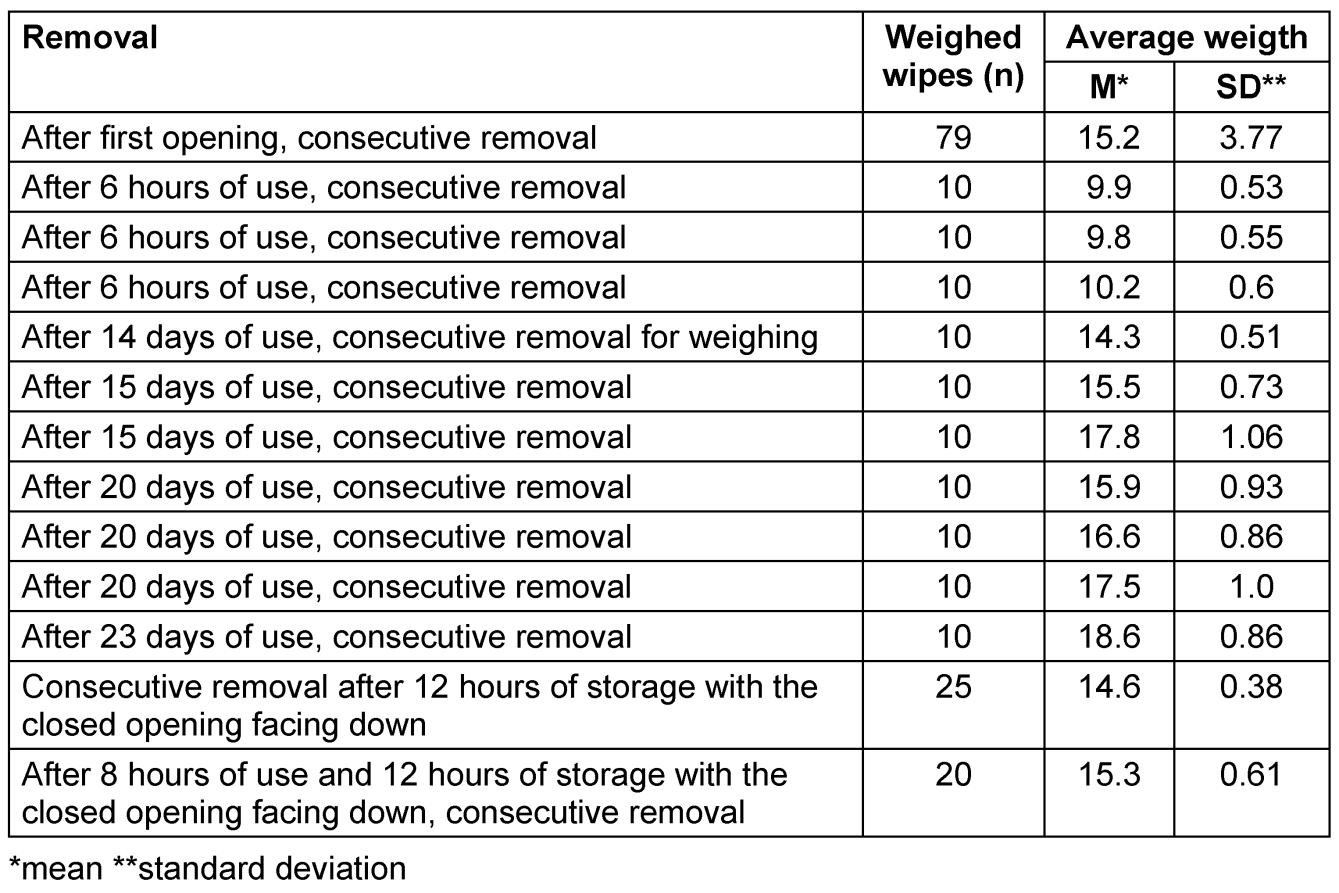
Average weights after first opening or after varying durations of use (horizontal storage; each package contents 80 wipes)

**Table 3:**
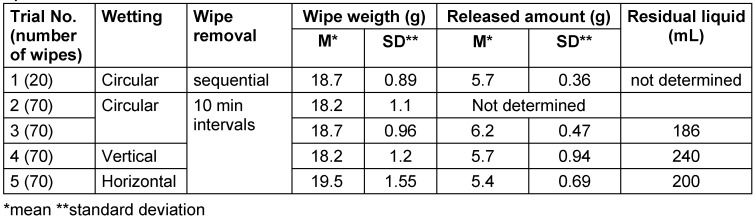
Weights of wipes and amounts of disinfectant solution released in wipe dispenser system 1

**Table 4:**
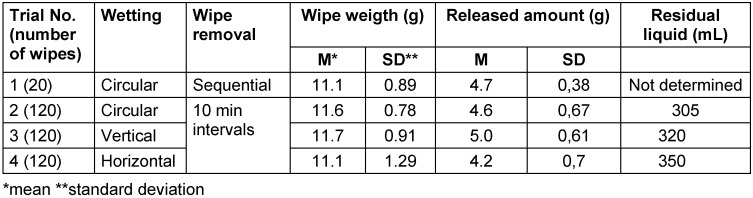
Weights of wipes and amounts of disinfectant solution released in wipe dispenser system 2

**Table 5:**
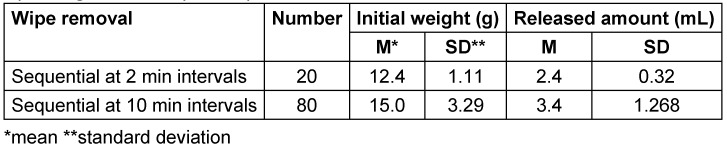
Weights of wipes and amounts of disinfectant solution released from wipes after opening the flow pack system

**Table 6:**
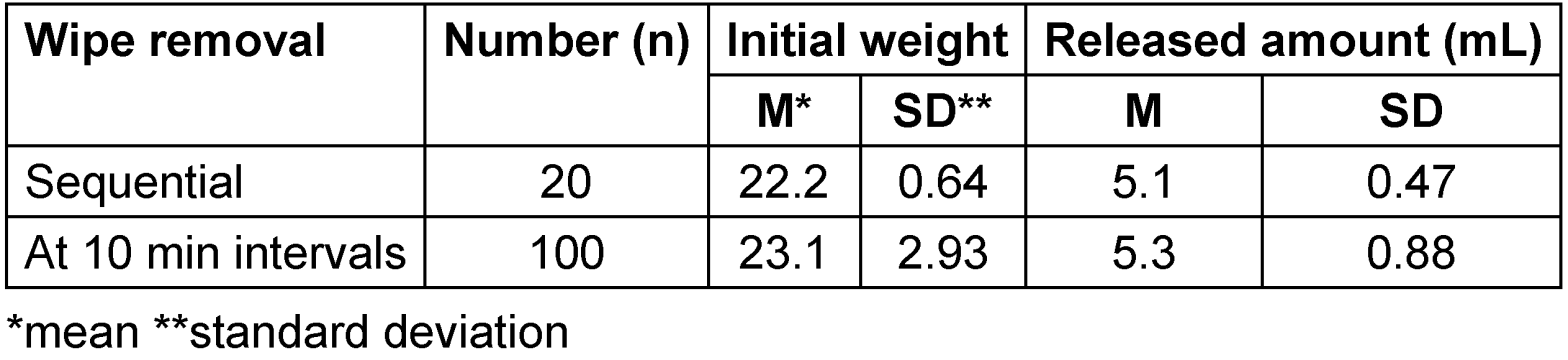
Weights of wipes and amounts of disinfectant solution released from wipes after opening the stand-up bag
